# Investigating the Association Between Family Socioeconomic Profile and Diabetes Control in Children: A Cross‐Sectional Study From Sudan

**DOI:** 10.1002/edm2.70014

**Published:** 2024-11-28

**Authors:** Hiba Ali Elzaki Hajomer, Osama Ahmed Elkhidir, Rooa Mohammed, Suodad Elhassan, Aya Abdelrahim, Yousra Ibrahim Abdallah Mohammed

**Affiliations:** ^1^ Department of Community Medicine National University Khartoum Khartoum State Sudan; ^2^ Faculty of Medicine University of Khartoum Khartoum Khartoum State Sudan

**Keywords:** diabetes control, glycaemic control, paediatric diabetes, socioeconomic status

## Abstract

**Aims:**

Diabetes mellitus is the most common endocrine disease in childhood which significantly impacts quality of life, morbidity and mortality. This study aimed to investigate the socioeconomic background of children with diabetes and their families and to assess its association with diabetes control.

**Methods:**

This cross‐sectional study was conducted in Khartoum State, Sudan, in 2018, at three outpatient diabetes clinics, using standardised questionnaires. We included 138 diabetic children (T1D and T2D) aged 2–18 years using systematic random sampling. Descriptive statistics, economic indicator and inferential statistics were applied using SPSS version 20. An HbA1C level of > 7.0% was set as the cut‐point for uncontrolled diabetes. A *p*‐value of < 0.05 was considered statistically significant.

**Results:**

The mean age of the children was 11.1 (3.64) years. Over half of the respondents were not covered by any insurance scheme and 73% lived in urban areas. Mothers had a mean age of 37.3 (7.8), with one‐fourth being illiterate, while fathers had a mean age of 45.4 (9.3). Twenty per cent of parents had higher education. Two‐thirds of the participants had their HbA1C levels checked within the last 3 months. The prevalence of uncontrolled diabetes was 78.0%. No statistically significant association was found between HbA1C levels and families' socioeconomic, demographic or clinical characteristics (*p* > 0.05).

**Conclusion:**

Neither children' nor parents' characteristics influenced HbA1C level. The high rate of uncontrolled diabetes (78.0%), and other countries showing better profiles of control, suggests the need for exploring other contributing factors. Insulin availability and lack of home glucose monitoring need to be considered. We recommend future prospective studies to consider these factors and use multiple HbA1C measures' average as a better indicator of diabetic control.

## Graphical abstract

This study investigates the socioeconomic background of children with diabetes in Sudan and its association with glycemic control. Despite a high prevalence of uncontrolled diabetes (78%), no significant link was found between HbA1C levels and socioeconomic or demographic factors.

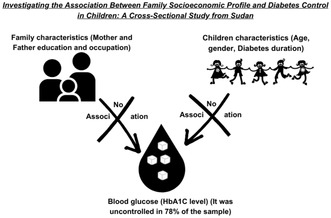

## 1 Introduction

Diabetes is a worldwide chronic metabolic disorder. It is the third most common paediatric endocrine disease [1], with approximately 60,000 children < 15 years of age with type 1 diabetes (T1D) in the Middle East and North Africa [2]. The global incidence of T1D has increased significantly in the last decades of the 20th century, in Europe an upturn was documented after 1955 in many countries, and rate of incidences continued in a linear increase afterwards [3]. The disease has a growing prevalence in Africa, in addition to a high prevalence of acute and long‐term complications, which are related to management challenges [4, 5]. In Sudan, a recent study estimated the overall incidence rate of T1D in children and adolescents to be 10.1/100,000, with a peak incidence age between 11 and 15 years [6]. The main dysfunction in diabetes is improper glucose utilisation that leads in the long run to serious side effects involving the heart, blood vessels, kidneys, eyes and nerves, It has two types: Type 1 (juvenile‐onset diabetes mellitus or insulin‐dependent diabetes mellitus) (T1D), which is due to the deficiency in production of insulin by the pancreas, and type 2 (insulin‐independent diabetes mellitus) (T2D), which is due to resistance to insulin action by the cells. T1D, in particular, has a polygenic susceptibility, HLA region on chromosome 6 is responsible for half of this genetic susceptibility [7, 8].

Lines of management differ according to the disease type; in T1D and as the exogenous insulin replacement did not achieve the optimum metabolic regulation, new technologies have emerged in addition to different insulin analogues (insulin pump and continuous glucose monitoring), which has contributed to improving the control of T1D over the last decades. According to the American Diabetes Association (ADA) standards of care, other types of treatments and practices are recommended in conjunction with standard insulin therapy; Pramlintide (amylin analogue) is sometimes indicated for patients on insulin therapy. Other drugs—that are primarily used for type 2—are being under consideration and studied regarding their usage in T1D (Metformin and the sodium–glucose cotransporter‐2 inhibitors). Other recommendations include self‐monitoring for glucose and continuous glucose monitoring; as they are proven to help optimising HbA1C levels. T2D mainly depends on oral hypoglycaemic drugs. Modification of lifestyle with Metformin is the most known first‐line treatment of this type. Some forms require combination therapies with or without introduction of insulin. However, the high prevalence of macro‐ and microvascular complications remains a concern. Novel drugs are being considered lately for their great improvement of glycaemic control, like inhibitors of the sodium–glucose cotransporter 2 (SGT‐2) [9, 10, 11, 12].

Diabetes glycaemic control test is an indirect test that is used to estimate glycaemia in the past 3 months, and it is important in assessing effectiveness and safety of the treatment. Diabetes glycaemic control is assessed by measuring glycated haemoglobin (HbA1C) in patients with T1D and T2D. Current guidelines advise a target of less than 6.5%–7.0% HbA1C [13, 14]. Many studies have stated that poor glycaemic control is a substantial risk factor for the progression of diabetic complications [15]; high HbA1C levels (> 53 mmol/mol, 7.17%) are associated with increased risk of both macrovascular and microvascular complications, irrespective of the treatment [16]. Furthermore, poor glycaemic control has been linked to higher healthcare costs [17, 18].

The socioeconomic status (SES) of an individual is defined as his/her social standing or class, operationalised as a combination of education, income and occupation [19]. According to studies demonstrating the association between SES, diabetes and its management, it has been found that in youth with T2D, low family income, parental education and high levels of juvenile stress are common [20, 21]; furthermore, the health outcomes in children and adolescents with T1D are greatly influenced by social and ethnic disparities [22], it is previously stated in the U.S. that diabetes effects on the adult populations—including rates of complications and mortality—differ according to many factors such as ethnicity and economic status [23], in addition, socioeconomic status is one among many other risk factors that could lead to sustained poor glycaemic control [24].

The increasing number of publications stating that the risk of diabetes, diabetes outcomes and control are largely affected by SES should be a huge concern, especially when considering that three out of every four people with diabetes live in low‐ and middle‐income countries (LMICs) [25].

Diabetes care in developing countries, particularly in African regions, requires more effort to address the disease profile of this subset and their social constraints, since this association may differ according to each society, and understanding of the association between different health determinants—such as socioeconomic status (SES)—and the control of diabetes will lead to more oriented interventions from authorities, and the further enhancement of the treatment outcome. There is an increased demand to evaluate this association in Sudan with little‐to‐no data. This study aimed to investigate the socioeconomic background of diabetic children and their families and the association between this profile and the control of diabetes in Khartoum State, Sudan.

## 2 Material and Methods

### 2.1 Study Design and Area

This is a cross‐sectional facility‐based study, conducted in public paediatric outpatient clinics for diabetes in Khartoum State, namely, Ahmed Gasim Hospital, Mohammed Elamin Hamid Hospital and Sudan childhood diabetes centre. Those centres cover the three major cities in Khartoum State that contain a population of more than 8 million residents. As for each one, Ahmed Gasim Hospital located in Bahri City, Khartoum North has public access. It contains outpatient clinics (emergency and refer). The outpatient clinic receives an average of 150 patients/day. Mohammed Elamin Hamid Hospital located in Omdurman City is a public teaching Hospital that mainly serves Omdurman residents, in addition to cases referred from Khartoum State. The outpatient clinic of this hospital receives an average of 300 patients/day. Sudan childhood diabetes centre is a specialised centre that was recently opened in south Khartoum area, it offers a multidisciplinary team including paediatric endocrinologists, nurses, dietitians, psychologists and social workers.

### 2.2 Study Sample

The study included 138 children between 2 and 18 years, diagnosed with diabetes at least 3 months before the time of the interview, followed up in a public specialised centre in Khartoum State, and whose parents accompanied them to the clinic, either father or mother as they were interviewed. Severely ill children and children whose parents refused to participate were excluded.

The study used probability sampling using systematic random sampling, the sample was distributed proportional to average patient frequency among the three facilities. Sample size was calculated using the following formula:

$$n={\left(\frac{1.96\times r}{y}\right)}^{2}$$

where *n* = sample size, equals 138; *r* = ratio of expected standard deviation to mean, equals (0.3); *y* = desired confidence interval width, equals (5%).

### 2.3 Data Collection Techniques

The data were collected using structured questionnaires about the socioeconomic profile and checklist for hospital care records to obtain clinical data. Data collectors were recruited from medical school of University of Khartoum. Training was conducted to six medical students in three sessions. A pilot study was conducted before starting the study. Twenty questionnaires were distributed and checked prior to data collection to test the feasibility, reliability and validity of the questionnaire and the expected human related and data optimisation problems at participating facilities. Informed consent was tested consequently, explanation was added and language that is more appropriate was adopted. The questionnaire was modified according to the feedback received from the pilot study (two questions were removed due to duplication and seven questions were rephrased to ensure reliability and the sequence of questions was revised). Mean time to fill the questionnaire was 20 ± 7 min, recruitment rate was 3–5 participants per centre per day and 90% of all daily eligible diabetic children can be recruited.

Epidemiologist, paediatric endocrinologist and statistician have validated the questionnaire. Sociodemographic data were collected regarding participants and their parents (age, gender and education), parents were asked if their children have any kind of private or government health insurance whether social, reimbursement scheme, monthly medical allowance, donor, family/community fund or other, data regarding clinical characteristics were obtained (type and duration of diabetes, type of treatment and other comorbidities), additional clinical history was obtained by asking about hospitalisation history, causes of hospitalisation related to diabetes (recurrent infections, diabetic ketoacidosis, renal and microvascular complications), hypoglycaemia episodes and ketonuria in the last 3 months. Data regarding control of diabetes were collected from the records; the last measurement of HbA1C in the last 3 months and parents' perception about their children's diabetes control were also recorded. Patient records were reviewed to confirm the diagnosis. School performance was measured subjectively based on parent's perception of their children's performance before and after diagnosis. Regarding socioeconomic characteristics of household: the primary income earner was specified, his/her education level, occupation, estimated personal take‐home earning per month, if the PIE had to quit or change jobs after the child's illness, parents were asked if they had to reduce the share of household income spent on food due to diabetes or if any of their children of/below school age work to finance costs due to their child's illness; residency details were obtained: place, number of rooms, house ownership, electrical and water supply, type of toilet facility.

### 2.4 Data Management and Analysis

Income details were transferred to the United States Dollar (USD) currency to satisfy international comparison using the official current exchange rate of the national bank. Data were coded, recorded in a spreadsheet, cleaned, managed and analysed using the statistical package for social science (SPSS) statistical package, version 20. Descriptive statistics considering median and mean values, and Fisher's exact test were used. Correlations were created, when appropriate.

## 3 Results

### 3.1 Sociodemographic Characteristics of Study Participants

The study sample included 138 diabetic children and their parents, with the mean age of children being 11.1 (3.64) years. The minimum and maximum age was 2 and 18 years respectively, and 72% are in primary school. Females represented 63%, and 73% lived in urban Khartoum State (Table 1).

**TABLE 1:** Participants' sociodemographic characteristics (*n* = 138).

Characteristic category	Subcategory	Count	Per cent
Age	2–4	9	6.5
	5–7	15	10.9
	8–12	60	43.5
	13–18	54	39.1
Residence	Urban	101	73.2
	Rural	37	26.8
Highest level of education of participant	Not attended	12	8.7
	Preschool	4	2.9
	Primary	99	71.7
	Secondary	23	16.7

### 3.2 Participants' Insurance Status and Clinical Characteristics

**FIGURE 1:**
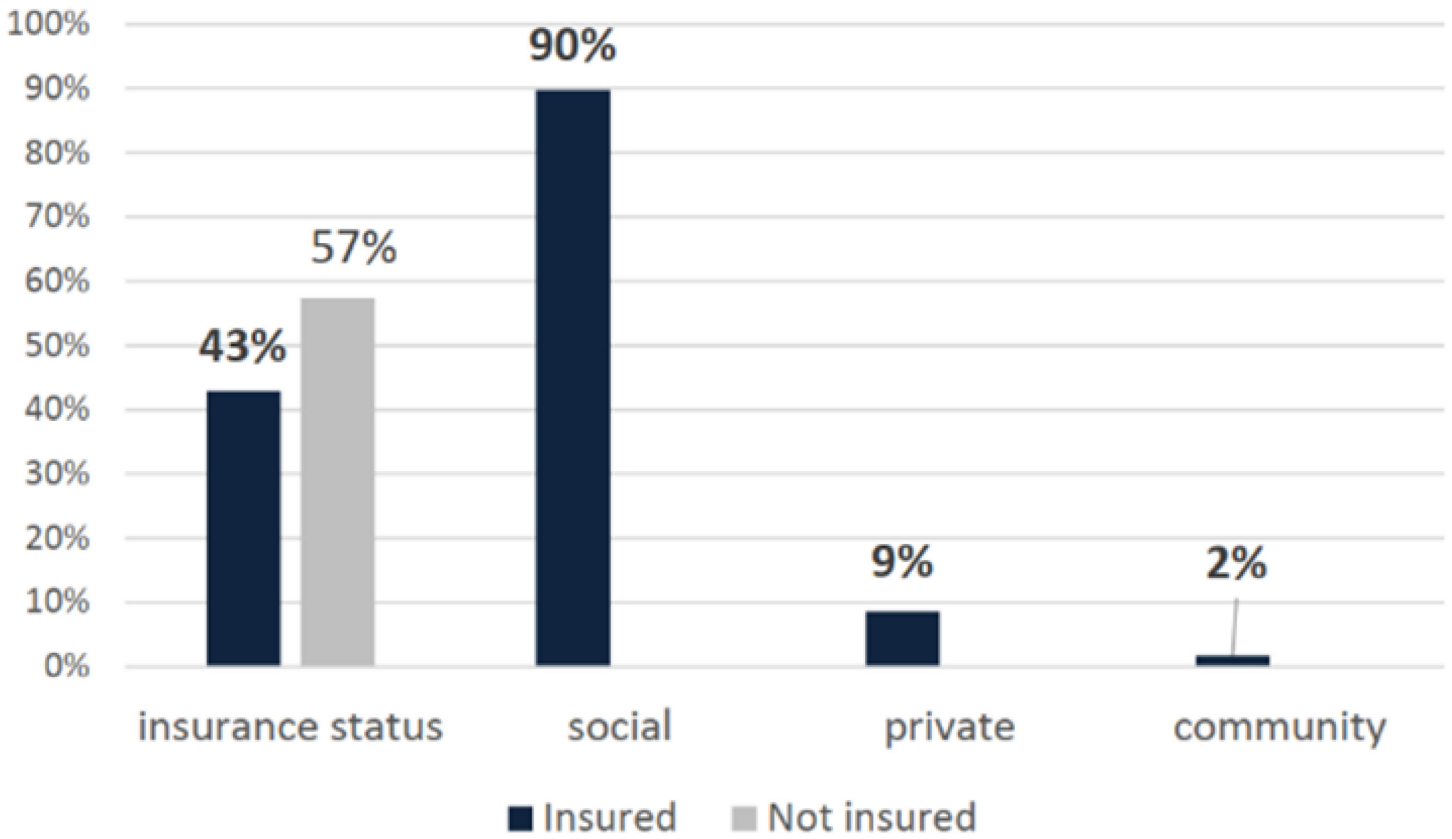
Participants' insurance status (*n* = 138).

Fifty‐seven per cent are not covered by any insurance scheme; among those who have insurance, the majority have social health insurance (provided by the government), followed by private health insurance and family/community insurance (Figure 1).

Type I diabetes was the most common (92%), and so insulin was the main treatment in the majority (94%) (Table 2). Fifty‐two per cent have had diabetes for 1–4 years, 90% of children had no other comorbidities apart from celiac and thyroid disease in 12 participants, and asthma and allergy in two participants (Table 2).

**TABLE 2:** Participants' clinical characteristics (*n* = 138).

Characteristic category	Subcategory	Count	Per cent
Type of diabetes	Type 1	127	92.0
	Type 2	10	7.2
	Other types	1	0.7
Treatment type	Insulin	130	94.2
	Oral	2	1.4
	Combined	6	4.3
Other disease status	Allergy	1	0.001
	Asthma	1	0.001
	Celiac	9	6.5
	Celiac and thyroid diseases	2	0.01
	No other diseases	125	90
Duration of diabetes	Less than 1 year	24	17.4
	1–4 years	72	52.2
	5 years or more	42	30.4
	Mean and SD	3.4 (2.8)	—

### 3.3 Parents' Socio‐Demographic Characteristics

The mothers' mean age was 37.3 (7.8) whereas the fathers' mean age was 45.4 (9.3) (Table 3). About one‐fourth of mothers were illiterate (higher than fathers, and lower than the illiteracy rate among females in Sudan by about 6%). Twenty per cent of the fathers and mothers were university graduates. 88.4% of the mothers were housewives, on the other hand, most fathers were working (94%), and about two‐thirds were free workers (Table 3).

**TABLE 3:** Mothers' (*n* = 138) and Fathers' (*n* = 138) sociodemographic characteristics.

Characteristic category	Subcategory	Mother		Father	
		Count	Per cent	Count	Per cent
Education	Illiterate	34	24.6	23	16.7
	Primary school	49	35.5	39	28.3
	Secondary school	27	19.6	46	33.3
	University	28	20.3	27	19.6
	Higher education	0	0	3	2.2
Occupation	Housewife/not working	122	88.4	8	5.8
	Officer	10	7.2	30	21.7
	Free work	6	4.3	87	63.0
	Merchant	0	0	7	5.1
	Farmer	0	0	1	0.7
	Other	0	0	5	3.6
Age	20–30	29	21.0	8	6.0
	31–40	73	52.9	40	30.1
	41–50	30	21.7	50	37.6
	51–60	6	4.3	28	21.1
	> 60	0	0	7	5.3
	Other	0	0	1	0.7
Diseases	—	22	16	20	14
Have children preschool age	Yes	117	84.8	—	—
	No	21	15.2	—	—

Approximately 16% and 14% of mothers and fathers, respectively, have other diseases, most of which are noncommunicable diseases including: diabetes and hypertension reflecting an extra burden on the families of diabetic children (Table 3).

### 3.4 Socioeconomic Characteristics of Household

The father was the primary income earner (PIE) in more than 80% of families. The mean income of PIE was 31,947 Sudanese pounds (SDG) per year (1775 USD/year) (SD = 43,239), whereas the median was 24,000 SDG (1333 USD), reflecting a high variability.

We also found that the weighted household income—after adjustment for household size—was more than six times lower than the household income (Table 4).

**TABLE 4:** Income of household in SDG per year and USD per year (*n* = 138).

Income	Subcategory	Mean	Median	SD	Max
PIE's income per year	SDG	31,947	24,000	43,239	288,000
	USD	1775	1333	2402	16,000
Total household income per year	SDG	33,459	24,000	43,552	288,000
	USD	1859	1333	2420	16,000
Weighted total household income^a^	SDG	5234	3429	7069	48,000
	USD	291	190	393	2667

Abbreviations: PIE, primary income earner; SDG, Sudanese pounds; USD, United States Dollar.

^a^ Weighted total household income, after adjustment to household size.

Most PIEs were educated; however, two‐thirds were only educated until primary or secondary school. The head of the household was the primary income earner in approximately 70% of families. The majority of PIEs were informal workers. Moreover, a quarter of parents were not financially independent. One‐third of children's parents did not own a house of residence: they either lived with the extended family or rented a house (Table 5).

**TABLE 5:** Socioeconomic characteristics of household (*n* = 138).

Characteristic category	Subcategory	Count	Per cent
Primary income earner in the household	Mother	8	5.8
	Father	121	87.7
	Extended family	4	2.9
	Son	3	2.2
	Daughter	1	0.7
	Other	1	0.7
Education of primary income earner	Not attended/Illiterate	20	14.5
	Primary	43	31.2
	Secondary	40	29.0
	Graduate	25	18.1
	Postgraduate	10	7.2
Education of head of household	Not attended/Illiterate	7	5.1
	Primary	9	6.5
	Secondary	15	10.9
	Graduate	1	0.7
	Postgraduate	10	7.2
Education of spouse of head of household	Not attended/Illiterate	33	24.6
	Primary	44	32.8
	Secondary	31	23.1
	Graduate/Certificate	26	19.4
Primary income earner employment status	Yes, formal work	48	34.8
	No, informal work	88	63.8
	Retired	1	0.7
	Other	1	0.7
Parents financially independent	Yes	104	75.4
	No	34	24.6
Current place of residence	Urban	99	72.3
	Urban slum	9	6.6
	Rural	29	21.2
Ownership of the house of residence	Yes	92	66.7
	No	46	33.3

Most households had public electricity and piped water lines, about 90% had their own toilets and 30% had flush toilets. The average number of rooms was 2.4 ± 0.9, whereas the average number of people living in the household was 7.3 ± 2.7 (Table 6).

**TABLE 6:** Amenities and housing characteristics of household (*n* = 138).

Characteristic category	Subcategory	Count	Per cent
Electricity supply	Public connection	120	87.0
	Other	1	0.7
	None	17	12.3
Source of drinking water	Lake/Pond/Dam/River	3	2.2
	Public well	36	26.1
	Private well/Bore hole	3	2.2
	Piped water	91	65.9
	Bottled water	5	3.6
Type of toilet facility	Shared pit toilet/Latrine	8	5.8
	Own pit toilet/Latrine	88	63.8
	Flush toilet	42	30.4
Rooms in the house	1 room	20	14.5
	2 rooms	56	40.6
	3 rooms	40	29.0
	4 or more rooms	22	15.9

In 15% of the families, one of the parents had to quit their income‐earning job to stay home and care for the child, and in 6%, primary income earners had to change jobs when the child became ill with diabetes. Furthermore, in 7% of the families, other children (siblings of diabetic children) of or below school age needed to work in order to finance the cost of treatment. Eighteen per cent of parents said that they had to reduce the share of household income spent on food due to diabetes. In addition, the school performance of children with diabetes decreased after illness.

### 3.5 Diabetes Control of Study Participants

Two‐thirds of participants had their HbA1C tested during the last 3 months. The rate of uncontrolled diabetes was 78.0%, with HbA1C equalling or more than 7.0 (Table 7). Also, 86.2% of participants did not have regular follow‐ups (Table 8); however, the perception of parents about diabetes control of their children differed from laboratory tests: the percentage of those who thought their children's diabetes control was good or very good (40%), was almost the same as that of those who thought it was poor or very poor (42%) and the remaining 18% perceived it as fair. Therefore, when comparing their perception with their level of HbA1C, there was a variation in the results. Additionally, 37% of patients had Ketonuria in the last 3 months (Table 7).

**TABLE 7:** Diabetes control of participants (*n* = 138).

Characteristic category	Subcategory	Count	Per cent
If HA1C checked during the last 3 months	Checked	94	68.1
	Not checked	44	31.9
HA1C level in the last time checked during the last 3 months	4%–5.6%	3	3.3
	5.7%–6.4%	4	4.4
	6.5%–7%	13	14.3
	More than 7%	71	78.0
Ketones in urine during the last 3 months	No ketones	87	63.0
	Ketones	51	37.0
Episodes of ketone in urine during the last 3 months	One	25	49.0
	Two	11	21.6
	Three	6	11.8
	Four	4	7.8
	Five	3	5.9
	Six	1	2.0
	Other (10 times)	1	2.0
Hypoglycaemia during last 3 months	No hypoglycaemia	73	52.9
	Hypoglycaemia	65	47.1
Episodes of hypoglycaemia during the last 3 months	One	14	21.5
	Two	13	20.0
	Three	12	18.5
	Four	10	15.4
	Five	6	9.2
	Six	1	1.5
	Seven	3	4.6
	Eight	1	1.5
	Ten	4	6.2
	About twenty	1	1.5

Abbreviation: HbA1C, haemoglobin A1C.

**TABLE 8:** Participants' management, follow‐up and hospitalisation (*n* = 138).

Characteristic category	Subcategory	Count	Per cent
Regular follow‐up	Yes	119	86.2
	No	19	13.8
Frequency of regular follow‐up	Once a week	3	2.5
	Every 2 weeks	5	4.2
	Monthly	73	61.3
	Every 2 months	37	31.1
	Other	1	0.8
Hospitalisation history during last year, due to causes related to diabetes	Yes	91	65.9
	No	47	34.1
The frequency of hospitalisation in the last year	1–3 times	81	89.0
	4–6 times	3	3.3
	7–9 times	5	5.5
	Other (15 times)	2	2.2

Regarding other markers of diabetic control, 47.1% confirmed the occurrence of hypoglycaemia during the past 3 months, of which 39.9% reported more than three hypoglycaemic episodes (mean = 3.7, median = 3) (Table 7). Most children (65.9%) had a history of hospitalisation due to causes related to diabetes (other than hypoglycaemia) during the last year, with a frequency of 1–3 times/last year for 89% of those who were hospitalised (Table 8).

### 3.6 Association Between Socioeconomic Profile and Control of Diabetes

We found that neither the children nor the parents' socioeconomic, clinical and demographic characteristics (age, sex, duration of illness, education and occupation) influenced HbA1C level as an indicator for diabetic control (*p* > 0.05, Fisher's exact test was used) (Table 9).

**TABLE 9:** Association between sociodemographic characteristics and control of diabetes (*n* = 138).

		HbA1C level								*p*
		≤ 5.6		5.7–6.4		6.5–7.0		> 7.0		
		Count	%	Count	%	Count	%	Count	%	
Gender	Male	1	3.1	3	9.4	4	12.5	24	75.0	0.394
	Female	2	3.4	1	1.7	9	15.3	47	79.7	
Duration of diabetes	< 1 year	1	6.7	1	6.7	2	13.3	11	73.3	0.675
	1–4.9 years	1	2.3	3	6.8	7	15.9	33	75.0	
	5 years and more	1	3.1	0	0.0	4	12.5	27	84.4	
Age of patient	2–4	0	0.0	1	20.0	0	0.0	4	80.0	0.337
	5–7	0	0.0	1	9.1	0	0.0	10	90.9	
	8–12	2	5.4	2	5.4	6	16.2	27	73.0	
	13–18	1	2.6	0	0.0	7	18.4	30	78.9	
Mothers' education	Illiterate	1	7.1	0	0.0	5	35.7	8	57.1	0.221
	Primary school	0	0.0	2	5.9	3	8.8	29	85.3	
	Secondary	0	0.0	1	5.6	2	11.1	15	83.3	
	University or higher education	2	8.0	1	4.0	3	12.0	19	76.0	
Mothers' occupation	Housewife/Nonworking	3	3.7	4	4.9	11	13.6	63	77.8	0.849
	Working	0	0.0	0	0.0	2	20.0	8	80.0	
Fathers' education	Illiterate	1	10.0	0	0.0	3	30.0	6	60.0	0.566
	Primary school	0	0.0	2	7.4	4	14.8	21	77.8	
	Secondary school	1	3.1	2	6.3	4	12.5	25	78.1	
	University or higher education	1	5.4	0	0.0	2	9.1	19	86.4	
Fathers' occupation	Not working	0	0.0	0	0.0	2	33.3	4	66.7	0.48
	Officer	2	8.7	1	4.3	1	4.3	19	82.6	
	Free work	1	1.8	2	3.6	10	18.2	42	76.4	
	Merchant	0	0.0	1	25.0	0	0.0	3	75.0	
	Other	0	0.0	0	0.0	0	0.0	3	100.0	

*Note: p*‐values > 0.05, Fisher's exact test was used.

## 4 Discussion

This cross‐sectional study describes the socioeconomic characteristics of diabetic children and their families, in public diabetes specialised clinics, and association of these factors with control of diabetes.

Due to the lack of sufficient data in Sudan, this is considered one of the novel studies that studied the link between socioeconomic characteristics and diabetes control. We found it important to investigate the impact of the continuously deteriorating economy of Sudan and the continuous decline of socioeconomic aspects of families living in recent years, as well as the effects of these acute changes on accessibility and affordability of healthcare services and behaviours of patients, especially those with chronic diseases, who may need certain diets, regular follow‐up and continuous treatment, such as diabetes patients.

The results showed that female gender represents about two‐thirds of diabetic children in the study population, whereas diabetes gender ratio was found to be roughly equal in children diagnosed < 15 years old according to a review conducted in the UK. A recent study in Sudan regarding epidemiology of childhood type 1 diabetes below the age of 15 showed no significant variation in prevalence among genders. This contradiction could be due to the ethnicity and other determinants in non‐European origin population [6, 26].

In families of our study participants, childhood diabetes caused many overt changes in family settings, economic consequences and social effects. In 5% of the families, one of the parents had to quit their income‐earning job to stay home and care for the child, primary income earners had to change jobs when the child became ill with diabetes in 6% and other family children of or below school age needed to work in order to finance the cost of treatment in 7% of the families, the amount of food consumed per month changed due to the child's illness (18%) and school performance of diabetic children decreased. These findings correspond to Elrayah H., where those with diabetes were significantly more likely to suffer from adverse social effects, such as being prevented from doing paid work or participation in education, both for themselves and their families. Moreover, these results are consistent with a study done in Spain in 2017 in children aged 15 or less, diabetes incurs considerable societal costs amongst paediatric patients, mainly informal care which represents the largest cost category, representing 83% of total cost [27, 28].

This study found that children and parents sociodemographic and socioeconomic characteristics do not influence control of diabetes (indicated by HbA1C level), these results differ from the results of a study that was held in the United States, showing significant association between socioeconomic profile and continuous HbA1C [29]. Another study in the US concluded that among youth with type 1 diabetes, individuals with lower SES were more likely to have high‐risk glycaemic control. Another study considered the racial disparities besides the SES, as lower SES and African American races were associated with higher levels of HbA1C across the age range [29, 30]. These differences could be attributed to other factors varying between Sudan and the United States (social and economic thematic disparities, racial variation and implementation of different current treatment modalities), suggesting that other factors might play a pivotal role in influencing diabetic control in Sudanese children. Regarding diabetes control in children, 40% of parents thought that diabetes control of their children was good or very good, and 34% thought that it was poor, or very poor (8%), but upon measuring HbA1c level, we found that the rate of uncontrolled diabetes was 78.0% (HbA1C > 7.0) (Table 7), which can be attributed to the fact that one‐third of them did not check their HbA1C level in the previous 3 months. Cognitive misperception might also contribute to below‐optimal control; a relatively high percentage of parents thought that their children's glycaemic control was good or very good, while it was not. This could reflect poor health education and a lack of information about the recommended and adequate levels of monitoring and control. Proper health education of caregivers can be one of the determinants of child's health, especially in chronic diseases, with continuous management and monitoring. This misperception might be as well, a barrier to glucose monitoring; other studies about parental perception and children diabetic control yielded that higher perceived competence about control was actually associated with lower levels of control and less frequent monitoring, and lower knowledge about diabetes to be associated with reduced efficacy of diabetic management in their children [31, 32]. A study in Sudan showed that the rate of poor glycaemic control was 78.9% with mean HbA1c (10.36 ± 2.14) [33], this percentage of poor glycaemic control is also comparable to another study in Nigeria in which 78.9% had HbA1C > 7.0, despite regular supply of insulin, suggesting that factors other than availability of insulin could play a role in glycaemic control and four important factors: young age, duration of diabetes < 2 years, young age at onset of diabetes and caregivers involvement in diabetes were identified as determinants of optimal glycaemic control. In general, younger ages are reported to have a different profile, as more severe metabolic decompensation is more common at the onset of diabetes in very young children [34, 35]; however, we did not find an association between age, gender or duration of diabetes and HbA1c levels. Another study in Tanzania has shown that family structure of diabetic children and the level of education of their caregivers were not significantly associated with glycaemic control [36].

These results suggest that there are other factors associated with the control and outcome of diabetes, a study found that social support is the single most important moderator of disease outcome especially in chronic diseases such as diabetes mellitus, mental illnesses, oncology and haematological diseases [37], which can be linked to increased adherence to therapy and required physical and dietary habits. A study in Egypt has shown that uncontrolled T1D among adolescents is associated with older age and psychiatric comorbidities, such as eating disorders, sadness, depression and anxiety. Behavioural eating habits and physical activity may partially explain the fluctuation in the diabetic control and its complications (especially hypoglycaemic episodes) [35, 38], also having health insurance was associated with 38% increase in the likelihood of seeking care, compared to noninsured [39], in our study, more than half of participants are not covered by any type of insurance, coverage by health insurance of study participants was low in comparison with the current level of coverage according to health insurance corporate of Khartoum State (75%; H.A.E.H., personal communication, 2021), this coverage level is not satisfactory to reach universal health coverage [40]. Variation in coverage level could be due to disparities in insurance coverage between rural and urban areas and between formal and informal sectors. Moreover, health insurance is essential for ensuring accessibility and affordability of health services, particularly with the increasing healthcare cost. Health Insurance can be a factor that determines healthcare‐seeking behaviour, and therefore, affects the outcome of diseases. With increasing healthcare costs in Sudan along with the economic inflation, noninsurance status exacerbates the economic burden that falls on families and communities [41]. Comparing that most of our participants suffer T1D, and incidences of ketonuria were 37%, besides an overall hospitalisation rate of about 66%. A cohort study investigating SES and DKA admission in youths with T1D had 34.6% of the sample admitted due to DKA and public insurance status was also associated with DKA admission (adjusted odds ratio [AOR], 2.71, 95% CI, 1.62–4.55) [42]. A recent study exploring the relation between the SES and insurance coverage for diabetes technology has concluded that the association between SES and insurance coverage and adverse outcomes was mainly mediated through treatment modalities related to diabetes technology use, suggesting that variation in diabetes control and disease outcome are linked to the upstream disparities in this regard [43]. It has also been found that across different ages (from children to the elderly), continuous glucose monitoring played a more prominent role in controlling diabetes in comparison to insulin pump usage, as the continuous glucose monitoring (CGM) usage without an insulin pump improved average HbA1C more than the pump without CGM; and pump versus injection use showed no difference in average HbA1C level in people with CGM [30]. Research investigating the implementation of current technologies came to a similar result; CGM in addition to other technologies such as using hybrid loop insulin delivery system is associated with a better glycaemic control [35]. Therefore, we believe that those factors might play a prominent role in affecting glycaemic control; especially when considering the high percentage of patients with uncontrolled diabetes (78.0%) (Table 7). Besides the different axes of variation (social, economic and demographic in both children and parents), none of them influenced HbA1C significantly.

In conclusion, high rate of poor diabetic control among Sudanese diabetic children can be attributed to many factors other than socioeconomic status. Data from other countries generally showed better profiles of glycaemic control, and further investigations are needed to determine predictors of glycaemic control in the Sudanese population and to develop management and authorities' intervention strategies.

One of the limitations of this study is recall bias; a single HbA1c measure was used although an average HbA1C over a period of time would be better because the records were not complete regarding this point and there was a recall bias about diabetes care and hospitalisation. More epidemiological data are needed, such as data on other diseases and the contribution of comorbidities and other factors to diabetes control. Therefore, future studies should adopt a prospective design. We recommend performing multiple measures over a period of time and using the average HbA1C as a more accurate indicator. We recommend not using only HbA1C to assess diabetic control, which can be affected by other conditions or comorbidities, insulin availability status and lack of home monitoring of glucose are other factors that should be considered in future studies.

## 5 Conclusion

This study found that neither children's nor parents' socioeconomic, demographic and clinical characteristics of the children (gender, age, duration of illness, parent's education and occupation) have affected the level of HbA1C (as an indicator for diabetes control) in Khartoum, Sudan. Rate of uncontrolled diabetes among this group was 78.0% (HbA1C > 7.0); other countries showed better control profiles. Poor glycaemic control can be attributed to many other factors, such as insulin availability and CGM. Those findings might help establish a clearer understanding of predictors and factors and their weight in influencing the control of the disease, and to develop interventional strategies accordingly.

## Author Contributions

H.A.E.H. and O.A.E. were involved in conceptualisation. O.A.E., H.A.E.H. and Y.I.A.M. developed the methodology. S.E., R.M., A.A. and Y.I.A.M. took part in investigations. O.A.E., S.E. and R.M. took part in data curation. O.A.E., H.A.E.H. and A.A. conducted the formal analysis. S.E., R.M., A.A. and Y.I.A.M. participated in the visualisation. Y.I.A.M., S.E., A.A. and R.M. were involved in writing – original draft. O.A.E. and H.A.E.H. were involved in writing – review and editing. H.A.E.H. was involved in supervision. All authors have read and given approval of the final version to be published, have agreed on the journal to which the article has been submitted and agreed to be accountable and responsible for all aspects of the work.

## Ethics Statement

Approval from Sudan Medical Specialisation Board, Khartoum State Ministry of Health research department and from selected hospitals was obtained. Participants had the right to withdraw at any time without any deprivation. Participants were not harmed by any means, there were no experimental protocols, confidentiality and questionnaire coding were conducted. Questionnaires were distributed and filled without interrupting health care providers.

## Consent

Informed consent was obtained from all children involved in the study and their accompanied caregivers, whether they were mother or father or both of them if they were both present.

## Conflicts of Interest

The authors declare no conflicts of interest.

## Data Availability

The data that supports the findings of this study are available from the corresponding author upon reasonable request.

## References

[1] A. T. Borchers , R. Uibo , and M. E. Gershwin , “The Geoepidemiology of Type 1 Diabetes,” Autoimmunity Reviews 9, no. 5 (2010): A355–A365, 10.1016/j.autrev.2009.12.003. 19969107

[2] H. Zayed , “Epidemiology of Diabetic Ketoacidosis in Arab Patients With Type 1 Diabetes: A Systematic Review,” International Journal of Clinical Practice 70, no. 3 (2016): 186–195, 10.1111/ijcp.12777. 26842462

[3] E. A. M. Gale , “The Rise of Childhood Type 1 Diabetes in the 20th Century,” Perspectives in Diabetes 51 (2002): 3353–3361. 10.2337/diabetes.51.12.3353 12453886

[4] A. O. Akanji , “Clinical Experience With Adolescent Diabetes in A Nigerian Teaching Hospital,” Journal of the National Medical Association 88 (1996): 101–105. 8776066 PMC2608019

[5] A. Elamin , M. I. A. Omer , K. Zein , and T. Tuvemo , “Epidemiology of Childhood Type I Diabetes in Sudan, 1987–1990,” Diabetes Care 15 (1992): 1556–1559. 1468286 10.2337/diacare.15.11.1556

[6] F. Saad , L. Mahmoud , A. Ali , I. Omer , and M. Abdullah , “Incidence and Prevalence of Type 1 Diabetes Mellitus in Children and Adolescents Aged 6 Months–19 Years in Khartoum State, Sudan,” Sudanese Journal of Paediatrics 20 (2020): 163–169, 10.24911/sjp.106-1587542600. 32817737 PMC7423301

[7] J. A. Noble , A. M. Valdes , M. D. Varney , et al., “HLA Class I and Genetic Susceptibility to Type 1 Diabetes: Results From the Type 1 Diabetes Genetics Consortium,” Diabetes 59, no. 11 (2010): 2972–2979, 10.2337/db10-0699. 20798335 PMC2963558

[8] P. Concannon , S. S. Rich , and G. T. Nepom , “Genetics of Type 1A Diabetes,” New England Journal of Medicine 360, no. 16 (2009): 1646–1654. 19369670 10.1056/NEJMra0808284

[9] I. B. Hirsch , “Realistic Expectations and Practical Use of Continuous Glucose Monitoring for the Endocrinologist,” Journal of Clinical Endocrinology and Metabolism 94, no. 7 (2009): 2232–2238, 10.1210/jc.2008-2625. 19383778

[10] J. J. Chamberlain , R. R. Kalyani , S. Leal , et al., “Treatment of Type 1 Diabetes: Synopsis of the 2017 American Diabetes Association Standards of Medical Care in Diabetes,” Annals of Internal Medicine 167, no. 7 (2017): 493–498, 10.7326/m17-1259. 28892816

[11] A. H. Barnett , A. A. Tahrani , C. Ord , J. Bailey , and S. Del Prato , “Management of Type 2 Diabetes: New and Future Developments in Treatment,” Lancet 378 (2011): 182–197, 10.1016/S0140. 21705062

[12] F. Ismail‐Beigi , “Glycemic Management of Type 2 Diabetes Mellitus,” New England Journal of Medicine 366, no. 14 (2012): 1319–1327. 22475595 10.1056/NEJMcp1013127

[13] D. M. Nathan , J. B. Buse , M. B. Davidson , et al., “Medical Management of Hyperglycemia in Type 2 Diabetes: A Consensus Algorithm for the Initiation and Adjustment of Therapy,” Diabetes Care 32, no. 1 (2009): 193–203, 10.2337/dc08-9025. 18945920 PMC2606813

[14] Y. Handelsman , J. I. Mechanick , L. Blonde , et al., “AACE Task Force for Developing Diabetes Comprehensive Care Plan. American Association of Clinical Endocrinologists Medical Guidelines for Clinical Practice for Developing a Diabetes Mellitus Comprehensive Care Plan,” Endocrine Practice 17, no. S2 (2011): 1–53, 10.4158/ep.17.s2.1. 21474420

[15] C. E. Koro , S. J. Bowlin , N. Bourgeois , and D. O. Fedder , “Glycemic Control From 1988 to 2000 Among U.S. Adults Diagnosed With Type 2 Diabetes A Preliminary Report,” (2004), http://diabetesjournals.org/care/article‐pdf/27/1/17/563607/zdc00104000017.pdf. 10.2337/diacare.27.1.17 14693960

[16] I. M. Stratton , A. I. Adler , A. W. Neil , et al., “Papers Association of Glycaemia With Macrovascular and Microvascular Complications of Type 2 Diabetes (UKPDS 35): Prospective Observational Study,” BMJ 321, no. 7258 (2000): 405–412. 10938048 10.1136/bmj.321.7258.405 PMC27454

[17] M. Mata‐Cases , M. Casajuana , J. Franch‐Nadal , et al., “Direct Medical Costs Attributable to Type 2 Diabetes Mellitus: A Population‐Based Study in Catalonia, Spain,” European Journal of Health Economics 17, no. 8 (2016): 1001–1010, 10.1007/s10198-015-0742-5. PMC5047944 26542160

[18] S. Shetty , K. Secnik , and A. K. Oglesby , “Relationship of Glycemic Control to Total Diabetes‐Related Costs for Managed Care Health Plan Members With Type 2 Diabetes,” Journal of Managed Care Pharmacy 11, no. 7 (2005): 559–564. 16137213 10.18553/jmcp.2005.11.7.559 PMC10437578

[19] S. Saegert , N. Adler , H. E. Bullock , and A. M. Cauce , “Report of the APA Task Force on Socioeconomic Status Help‐Seeking Behavior and Barriers Among Youth and Young Adults View Project Living Wage for Factory Workers‐DR View Project,” https://www.researchgate.net/publication/275100689.

[20] A. M. Butler , “Social Determinants of Health and Racial/Ethnic Disparities in Type 2 Diabetes in Youth,” Current Diabetes Reports 17, no. 8 (2017): 60, 10.1007/s11892-017-0885-0. 28664253 PMC5728112

[21] I. Kawachi , N. Daniels , and D. E. Robinson , “Health Disparities by Race and Class: Why Both Matter,” Health Affairs 24, no. 2 (2005): 343–352, 10.1377/hlthaff.24.2.343. 15757918

[22] A. P. Borschuk and R. S. Everhart , “Health Disparities Among Youth With Type 1 Diabetes: A Systematic Review of the Current Literature,” Families, Systems & Health 33, no. 3 (2015): 297–313, 10.1037/fsh0000134. 25984737

[23] S. H. Golden , N. Maruthur , N. Mathioudakis , et al., “The Case for Diabetes Population Health Improvement: Evidence‐Based Programming for Population Outcomes in Diabetes,” Current Diabetes Reports 17, no. 7 (2017): 51, 10.1007/s11892-017-0875-2. 28567711 PMC5553206

[24] D. T. Juarez , T. Sentell , S. Tokumaru , R. Goo , J. W. Davis , and M. M. Mau , “Factors Associated With Poor Glycemic Control or Wide Glycemic Variability Among Diabetes Patients in Hawaii, 2006–2009,” Preventing Chronic Disease 9, no. 9 (2012): 120065, 10.5888/pcd9.120065. 23017247 PMC3475506

[25] International Diabetes Federation , “Diabetes Atlas 2021,” 10th ed. (2021), https://diabetesatlas.org/atlas/tenth‐edition/. 35914061

[26] E. Gale and K. Gillespie , “Diabetes and Gender,” Diabetologia 44, no. 1 (2001): 3–15. 11206408 10.1007/s001250051573

[27] H. Elrayah , M. Eltom , A. Bedri , A. Belal , H. Rosling , and C. G. Östenson , “Economic Burden on Families of Childhood Type 1 Diabetes in Urban Sudan,” Diabetes Research and Clinical Practice 70, no. 2 (2005): 159–165, 10.1016/j.diabres.2005.03.034. 15919129

[28] J. López‐Bastida , L. M. Peña‐Longobardo , I. Aranda‐Reneo , E. Tizzano , M. Sefton , and J. Oliva‐Moreno , “Social/Economic Costs and Health‐Related Quality of Life in Patients With Spinal Muscular Atrophy (SMA) in Spain,” Orphanet Journal of Rare Diseases 12, no. 1 (2017): 141, 10.1186/s13023-017-0695-0. 28821278 PMC5563035

[29] M. W. Sutherland , X. Ma , B. A. Reboussin , et al., “Socioeconomic Position Is Associated With Glycemic Control in Youth and Young Adults With Type 1 Diabetes,” Pediatric Diabetes 21, no. 8 (2020): 1412–1420, 10.1111/pedi.13112. 32902080 PMC8054269

[30] K. M. Miller , R. W. Beck , N. C. Foster , and D. M. Maahs , “HbA1c Levels in Type 1 Diabetes From Early Childhood to Older Adults: A Deeper Dive Into the Influence of Technology and Socioeconomic Status on HbA1c in the T1D Exchange Clinic Registry Findings,” Diabetes Technology & Therapeutics 22, no. 9 (2020): 645–650, 10.1089/dia.2019.0393. 31905008 PMC7640747

[31] H. M. Pattison , “The Relationship Between Parental Perceptions of Diabetes and Glycaemic Control,” Archives of Disease in Childhood 91, no. 6 (2006): 487–490, 10.1136/adc.2004.061416. 16246854 PMC2082788

[32] A. Lohan , A. Morawska , and A. Mitchell , “Associations Between Parental Factors and Child Diabetes‐Management–Related Behaviors,” Journal of Developmental and Behavioral Pediatrics 38, no. 5 (2017): 330–338, 10.1097/dbp.0000000000000447. 28520634

[33] N. A. H. Abdelseed , “Glycemic Control and Knowledge Among Children and Adolescents With Type 1 Diabetes Mellitus; A Cross‐Sectional Study,” Journal of Scientific Research in Medical and Biological Sciences 2, no. 1 (2021): 1–9, 10.47631/jsrmbs.v2i1.149.

[34] C. F. Ogugua , U. N. Chikani , C. Y. Okiche , and U. M. Ibekwe , “Sociodemographic Determinants of Glycaemic Control Among Children With Type 1 Diabetes in South Eastern Nigeria,” Pan African Medical Journal 38 (2021): 250, 10.11604/pamj.2021.38.250.19790. 34104298 PMC8164434

[35] M. Nevo‐Shenker , M. Phillip , R. Nimri , and S. Shalitin , “Type 1 Diabetes Mellitus Management in Young Children: Implementation of Current Technologies,” Pediatric Research 87, no. 4 (2020): 624–629, 10.1038/s41390-019-0665-4. 31715623

[36] M. Noorani , K. Ramaiya , and K. Manji , “Glycaemic Control in Type 1 Diabetes Mellitus Among Children and Adolescents in a Resource Limited Setting in Dar es Salaam – Tanzania,” BMC Endocrine Disorders 16, no. 1 (2016): 29, 10.1186/s12902-016-0113-y. 27246505 PMC4886407

[37] A. Goldberg and K. S. Rickler , “The Role of Family Caregivers for People With Chronic Illness,” Medicine and Health, Rhode Island 94, no. 2 (2011): 41–42. 21456374

[38] S. A. Elhabashy , E. M. M. Sherif , N. Y. Salah , M. A. E. A. Elkader , and D. A. H. Youssef , “Uncontrolled Type 1 Diabetes Among Egyptian Adolescents; Risk Determinants and Clinical Outcomes,” Diabetes Epidemiology and Management 6 (2022): 100051, 10.1016/j.deman.2022.100051.

[39] I. Baloul and M. Dahlui , “Does Health Insurance Improve Utilisation of Healthcare Services, for Chronic Illnesses in Sudan?,” BMC Health Services Research 14, no. S2 (2014): P6, 10.1186/1472-6963-14-s2-p6.

[40] A. M. A. Salim and F. H. M. Hamed , “Exploring Health Insurance Services in Sudan From the Perspectives of Insurers,” SAGE Open Medicine 6 (2018): 205031211775229, 10.1177/2050312117752298. PMC5768257 29348914

[41] R. M. Weinick , M. E. Weigers , and J. W. Cohen , “Children's Health Insurance, Access to Care, and Health Status: New Findings,” Health Affairs 17, no. 2 (1998): 127–136, 10.1377/hlthaff.17.2.127. 9558790

[42] A. R. Maxwell , N. H. Y. Jones , S. Taylor , et al., “Socioeconomic and Racial Disparities in Diabetic Ketoacidosis Admissions in Youth With Type 1 Diabetes,” Journal of Hospital Medicine 16, no. 9 (2021): 517–523, 10.12788/jhm.3664. 34424192

[43] E. M. Everett and L. E. Wisk , “Relationships Between Socioeconomic Status, Insurance Coverage for Diabetes Technology and Adverse Health in Patients With Type 1 Diabetes,” Journal of Diabetes Science and Technology 16, no. 4 (2022): 825–833, 10.1177/19322968211050649. 34632819 PMC9264432

